# Spurious Quinine

**Published:** 1849-05

**Authors:** 


*Spurious Quinine.*—A distinguished chemist addressed us a note, last week, asking if it would not be advisable to let our medical readers know that they must be careful in purchasing sulphate of quinine, as there is a large quantity in the market shamefully adulterated, to the extent of twenty-five per cent. by analysis. So it seems that the new drug inspection laws cannot prevent all the fraudulent schemes in the matter of medicine. This one item of information shows very clearly that our apprehensions, some months since, were well founded, that the cheating in drugs would hereafter be accomplished at home instead of abroad. The advantages of changing the system, by Congress, are, that our own unprincipled dealers get a double profit. Formerly they sold the spurious articles already prepared; now they perform the adulterating manipulations themselves, and thus conduct a thriving business. An active demand for sulphate of quinine has grown out of the California epidemic—the fact being notorious that intermittent fevers are one of the accompaniments of gold hunting. This circumstance, therefore, has very likely induced individuals to exert themselves with activity, to supply the demand, so that all who wish may carry a bottle to the new El Dorado. One of the most lamentable features in this abominable deception, is, that there are no legal means of arresting the sale. Even arsenic and prussic acid may be sold at the corners of the streets, by apple-boys, for ought we know to the contrary, without trespassing upon any law or ordinance existing in this ancient Commonwealth. Notwithstanding the national law, therefore, in regard to drugs and medicines, the ingenuity which has been developed is working immense mischief.—*Boston Med. and Sur. Journal.*

